# Cost-Effective Care Coordination for People With Dementia at Home

**DOI:** 10.1093/geroni/igz051

**Published:** 2020-01-01

**Authors:** Amber Willink, Karen Davis, Deirdre M Johnston, Betty Black, Melissa Reuland, Ian Stockwell, Halima Amjad, Constantine G Lyketsos, Quincy M Samus

**Affiliations:** 1 Department of Health Policy and Management, Johns Hopkins Bloomberg School of Public Health, Baltimore, Maryland; 2 Department of Psychiatry and Behavioral Sciences, Johns Hopkins University School of Medicine, Baltimore, Maryland; 3 The Hilltop Institute, University of Maryland Baltimore Country, Baltimore, Maryland; 4 Department of Medicine, Johns Hopkins University School of Medicine, Baltimore, Maryland

**Keywords:** Care management, Cognitive impairment, Community health worker, Medicaid, Medicare

## Abstract

**Background and Objectives:**

People with dementia (PWD) represent some of the highest-need and highest-cost individuals living in the community. Maximizing Independence (MIND) at Home is a potentially cost-effective and scalable home-based dementia care coordination program that uses trained, nonclinical community workers as the primary contact between the PWD and their care partner, supported by a multidisciplinary clinical team with expertise in dementia care.

**Research Design and Methods:**

Cost of care management services based on actual time spent by care management personnel over first 12 months of MIND at Home intervention was calculated for 342 MIND at Home recipients from Baltimore, Maryland and surrounding areas participating in a Centers for Medicare and Medicaid Services (CMS) funded Health Care Innovation Award demonstration project. Difference-in-differences analysis of claims-based Medicaid spending of 120 dually-eligible MIND at Home participants with their propensity score matched comparison group (*n* = 360).

**Results:**

The average cost per enrollee per month was $110, or $1,320 per annum. Medicaid expenditures of dually-eligible participants grew 1.12 percentage points per quarter more slowly than that of the matched comparison group. Most savings came from slower growth in inpatient and long-term nursing home use. Net of the cost of the 5-year MIND at Home intervention, 5-year Medicaid savings are estimated at $7,052 per beneficiary, a 1.12-fold return on investment.

**Discussion and Implications:**

Managed care plans with the flexibility to engage community health workers could benefit from a low-cost, high-touch intervention to meet the needs of enrollees with dementia. Limitations for using and reimbursing community health workers exist in Medicare fee-for-service, which CMS should address to maximize benefit for PWD.

**Translational Significance**

Maximizing Independence (MIND) at Home is a cost-effective and scalable home-based dementia care coordination program that uses trained, nonclinical community workers as the primary contact, supported by a multidisciplinary clinical team with expertise in dementia care. Evaluation of Medicaid spending show a slower increase in spending over time among MIND at Home participants compared to the matched comparison group and a redistribution of spending away from inpatient and institutional services toward home and community-based services. Managed care plans, particularly those enrolling Medicaid beneficiaries, are well positioned to implement this low-cost, high-touch, dementia care coordination program.

## Background and Objectives

In the United States, two-thirds of the 5.4 million people with dementia (PWD) live in their homes in the community, reflecting the growing desire of Americans to age in place (Kasper, Freedman, Spillman, & Wolff, 2015). The complexity of needs of PWD including medical, behavioral, and social needs, make it one of the most expensive chronic conditions in the United States (Hurd, Martorell, Delavande, Mullen, & Langa, 2013), and a strong candidate for improving care and reducing costs through care management support (Samus et al., 2018a). Navigating fragmented systems of health care, long-term services and supports, and community or social supports to meet the diverse needs of PWD is challenging. Currently, family and unpaid caregivers provide the bulk of support PWD receive to continue living in their homes (Kasper et al., 2015; Willink, Davis, Mulcahy, & Wolff, 2017) Caregiver burden, measured objectively (count of hours) or subjectively (caregiver’s perception of impact on themselves), is associated with poorer outcomes among people living with dementia (Gallagher et al., 2011; Kuzuya et al., 2011; Tanner et al., 2015). Best practice models of dementia care recognize and support the contributions of caregivers to the health and well-being of the person with dementia across the care continuum (Callahan et al., 2014).

Given most PWD live in their homes, many models are now concentrating on the home setting as an effective way to identify and address a broader set of unmet needs (e.g., social and environmental determinants of health) and provide more comprehensive “family-centered” support. This approach aligns with value-based care principles and bridges the health care with community setting (Samus et al., 2018a). While many of these models operate with a team-based approach, they vary dramatically in their composition. The position of care coordinator (also referred to as care manager, service coordinator) is frequently allocated to a nurse or social worker (Reilly et al., 2015; Tan, Jennings, & Reuben, 2014). Use of a health care or social service professional in this role raises important questions about sustainability and cost-effectiveness of such programs given the projected increase in prevalence of the older population living with dementia and workforce needs (McWilliams, 2016).

Innovations in workforce development and community-based service delivery capabilities are likely a potent tool for improving home-based dementia care. A new category of providers that include community health workers, care navigators, health coaches, and peer mentors are being recruited to work with clinicians and other health care providers to address gaps in care and support in the existing delivery system. These community workers are usually laypersons from the community, trained by health care organizations to support patients in a variety of roles (Kangovi et al., 2018). Evidence from many chronic disease based interventions have shown community workers to be effective in improving outcomes across various conditions including diabetes, cancer, cardiovascular disease, and asthma (Heisler, Vijan, Makki, & Piette, 2010; Kangovi et al., 2017; Lohr, Ingram, Nuñez, Reinschmidt, & Carvajal, 2018).

Drawing from these experiences, the MIND at Home program was developed as a comprehensive, home-based care coordination program for PWD and their care partners (Samus et al., 2017). Like other home-based models of dementia care, it follows a team-based approach; however, the team consists of trained, nonlicensed community workers as the front-line memory care coordinators (MCC) supported by a core clinical team of geriatric psychiatrists and registered nurses (RNs) who specialize in dementia care (Samus et al., 2017). The model can be augmented, based on case-mix and service recipient needs, to include other health professionals such as occupational therapists (Samus et al., 2018b).

In a pilot randomized control trial (RCT) of the MIND at Home intervention, those who received the program experienced significant delays in time to transition from home or death (Samus et al., 2014), increased use of dementia-related outpatient medical care and nonmedical community services (Amjad et al., 2018), better quality of life (Samus et al., 2014), fewer unmet care needs related to home safety and legal/advance care planning (Samus et al., 2014), and modest decreases in the number of hours caregivers spent with participants compared to a similar group (Tanner et al., 2015). In this analysis, we examine the cost of providing this dementia care coordination program to older adults with dementia and their care partners in the greater Baltimore and Maryland suburban District of Columbia region as part of a Centers for Medicare and Medicaid Innovation (CMMI)-funded Health Care Innovations Award (HCIA) (Samus et al., 2017). We also present the pre- and posthealth and long-term care spending data comparing those who received the MIND at Home intervention to propensity score the matched comparison group. We then estimate the return on investment of this model to the Medicaid program.

## Research Design and Methods

This is a prospective, quasi-experimental intervention trial design to evaluate the impact of a comprehensive home-based dementia care coordination intervention (Samus et al., 2017). This analysis of the MIND at Home intervention consists of two parts: (a) a calculation of the intervention delivery cost (cost of care management) for the full intervention sample (*n* = 342); and (b) Medicaid cost savings for a subgroup of the intervention (*n* = 120) compared to a matched control group (*n* = 360) for which administrative claims data was available. This study was approved by the Johns Hopkins Medicine Institutional Review Board and the Maryland Department of Health and Mental Hygiene Institutional Review Board.

### Part 1: Cost of Care Management

#### Participants

Three-hundred and forty-two (256 dual eligible, 86 Medicare-only) PWD living at home in the Greater Baltimore/Washington region in Maryland were enrolled in the project between March 2015 and October 2016 through a multicomponent, community-based outreach campaign that included referrals from community organizations, health care providers, local and state Medicaid waiver programs, health departments, and broad outreach via community-based events, advertising and media publicity. Eligible participants met all-cause dementia diagnostic criteria, were dually eligible for Medicaid and Medicare benefits or were Medicare-only, and were living at home in the community. Participants received MIND at Home for a median of 16 months (interquartile range 13–18 months). Dual eligibility in this study refers to an individual being eligible for both the Medicare (older adults) and Medicaid (low-income) program in the United States (inclusive of both partial and full duals). Participants were not required to have a family care partner (i.e., a person who provides nonpaid assistance in one or more instrumental activities of daily living) but were required to have a knowledgeable study partner to provide proxy information (Samus et al., 2017).

#### Intervention

The intervention and design for this study has been previously detailed by the authors (Samus et al., 2017). MIND at Home is a comprehensive, home-based dementia care coordination model that systematically assesses and addresses a broad range of unmet dementia-related care needs for PWD and care partners that place older adults at risk for health disparities, high healthcare costs, poor clinical outcomes, poor quality of life, and caregiver burden. MIND at Home is implemented by an interdisciplinary team that draws on and synthesizes the expertise and experience of trained nonclinical community workers (i.e., MCC), nurses, physicians (i.e., geriatric psychiatrists), and occupational therapists. The intervention uses a traditional care management process (i.e., comprehensive assessment, individualized care planning, implementation of the care plan, monitoring the impact over time, and reassessment and revising the care plan over time) to identify and address 13 broad care need domains (59 individual needs) for persons with dementia and the care partner. The intervention compliments but does not supplant existing Medicaid covered services such as home and community-based services (HCBS). For example, an MCC might identify transportation challenges as a barrier to accessing medical care and would link the PWD with the Medicaid funded transportation support or might help identify and support the development of a stronger informal support network to reduce social isolation. Further, by virtue of the family-centric focus on a broad range of dementia-related needs, MCCs provide a great deal of dementia education, skills coaching, an emotional support to informal family caregivers, whose needs are not typically directly addressed in HCBS.

MCCs were the primary interventionists responsible for cases and were hired through two partner community-based organizations, Johns Hopkins Home Health Care Group (JHHCG) and Jewish Community Services (JCS). The RNs and geriatric psychiatrists, employed by Johns Hopkins University School of Medicine, supported and mentored MCCs. Each partner organization (JCS, JHHCG), employed a clinical site supervisor (i.e., Doctoral Level Physical therapist at JHHCG, Licensed Clinical Social Worker at JCS) who provided day-to-day supervision of the MCCs, conducted quality improvement reviews, participated in the team-based mentorship meetings, and supported other MCC. An optimal caseload per one FTE MCC is 40–50 dyads (i.e., persons with dementia and family caregiver). In addition to participating in a joint initial in-home assessment and individualized care plan development for the PWD and care partners with the team nurse, MCCs were responsible for implementation and monitoring of the care plan and various care strategies indicated (e.g., provided PWD and care partners resource referrals, help with long-term care services and supports navigation and coordination, dementia education, behavior management skills education and training, emotional support, problem-solving strategies) (Samus et al., 2018b). Ongoing structured support and mentoring from the clinical team members are then provided via weekly team-based collaborative sessions, supplemented with in-person, phone, and virtual support as needed. The type and frequency of coordinator involvement with the persons with dementia and family is individualized and driven by need-level, care plan, and family preference. MCC aim to have a contact every 30 days, at minimum. All team members complete a comprehensive 40-hr training program on dementia followed by a practicum in the field.

MCCs are typically bachelor’s prepared (or equivalent) individuals who may come from a variety of backgrounds, with various technical skills and work experiences. The most essential qualifications for MCCs are that they have excellent communication and interpersonal skills (e.g., good customer service), an intrinsic desire to work with older individuals, demonstrated self-management (e.g., time management, adaptability) abilities, are problem solvers, work well collaboratively, and are creative and organized.

#### Cost analysis of care management

Total cost of care management per participant was calculated by summing the hourly cost of MIND at Home staff type (base salary plus fringe benefits) multiplied by time (minutes) each staff type spent providing care coordination related activities. All time spent on care management for participants (including preparation, documentation, encounters, and consultation/supervision/ quality control) were documented in the cloud-based electronic Dementia Care Management System (DCMS 2.0). In addition to the costs of care, costs accrued due to travel to and from the participants house (including time and mileage) was documented as well as additional supplies required to provide care management. While best efforts were made to ensure accurate documentation of time on the project, an internal survey conducted of all MCCs suggested that an average of 30 min of time spent coordinating care per dyad per month was not documented in the DCMS 2.0 record system. To provide a conservative estimate of the costs of providing care coordination, we have adjusted our estimates to include an additional 30 min of time per dyad per month to each MCC.

### Part 2: Medicaid Cost Savings

#### Participants

One hundred and twenty of the 342 participants are included in the analysis of Medicaid expenditures. This excludes Medicare-only MIND at Home participants (*n* = 86) and Medicare Advantage beneficiaries who are not dually enrolled in Medicaid (*n* = 95), as this analysis is restricted to Medicaid data currently available to us. Medicare beneficiaries dually-eligible for the Medicaid program but are classified as Supplemental Low-income Medicare Beneficiaries (SLMB), who only receive premium assistance are also excluded as no Medicaid claims data are available for this group (*n* = 37). Of the 120 participants, 70 are full duals and 50 are Qualified Medicare Beneficiaries (QMBs). For full duals, Medicaid covers premiums and cost-sharing related to their Medicare health coverage, and also covers additional services including home and community-based services. For QMBs, Medicaid only covers the premiums and cost-sharing related to their insurance, we therefore do not have information on HCBS or long-term nursing home use unless they subsequently become full duals. QMBs can become full duals if they meet additional financial or medically needy criteria. Some QMBs in the participant and comparison groups spent down into full dual eligible status over time and are included as their Medicaid claims data became available. Further, the analysis excludes individuals who had no claims over the follow-up period, as well as those with inpatient hospital stays longer than 60 days and one participant with an extraordinary medical procedure unrelated to dementia (transplantation) (*n* = 4).

#### Comparison group

A match was drawn for all study participants with comparison Medicare beneficiaries on a 1:3 basis using a combination of Medicare and Maryland Medicaid data using nearest neighbor propensity score matching. For this analysis of cost savings, 120 participants were matched to 360 individuals in a comparison group using the following process. The Medicare comparison group includes a diagnosis of Alzheimer’s or other dementia in claims records. The case–control matching using Medicare files is based on baseline dual eligibility status, age, race, gender, prior health care and long-term care utilization, number of chronic conditions, and combined Medicare and Medicaid expenditures from a 2-year lookback period. Individuals from the treatment group who had a pre-March 2015 diagnosis were matched to comparison individuals who also had a pre-March 2015 diagnosis, while the rest of the treatment group was matched to the entire comparison pool, regardless of diagnosis date. This is meant to control for the stage of dementia that the individuals were experiencing as of the assignment date.

With the exception of age and number of chronic conditions, the propensity scoring matching reported limited statistical significance of both the goodness-of-fit and coefficient estimates. This suggests that, for the most part, the likelihood of MIND at Home participation was consistent across demographics and service utilization, likely due to the mandatory pre-matching criteria of an Alzheimer’s or dementia diagnosis already limiting the comparison pool to similar high-need individuals. Supplementary Table 1 provides information on the balance between intervention and control group before and after matching. Despite the lack of statistical significance, the matching process did work in aligning the balance of the participant and comparison groups.

### Analysis of Medicaid cost savings

Medicaid savings are estimated by using a difference-in-difference analysis. We examine the difference between participant’s and the comparison group’s Medicaid expenditures 2 years pre-MIND at Home intervention and five quarters (15 months) postintervention for dually-eligible beneficiaries. Enrollment of participants occurred over a period from March 2015 to October 2016. For example, for those enrolling in July 2015, the pre-MIND at Home intervention data for the participant and matched control would be for 2 years from July 2013 to June 2015. The postintervention period is five quarters following enrollment, with Medicaid data collection ending in October 2017. Medicaid spending data includes the cost-sharing (coinsurance, deductibles, copayments) for Medicare covered services (e.g., inpatient, outpatient, physician spending), and total spending for services not covered by Medicare including home and community-based services and long-term facility care. The analysis does not include an estimate of Medicare savings. Importantly, there are no estimated direct Medicaid savings for the nonduals, although if MIND at Home participation decreases the risk of dual eligibility there would be indirect Medicaid savings.

Using the results from the cost of care management analysis (part 1) as well as the difference-in-difference analysis of Medicaid spending over time (part 2), we estimate a return on investment over a 5-year time-period. This return on investment analysis assumes costs of the intervention continue at the same rate over the 5-year period and that the trends in spending for participants and the comparison group continue over the 5-year period.

## Results

Intervention participants (*n* = 342) were an average age of 80.7 (standard deviation [*SD*] = 9.8); mostly (75%) female; and were racially diverse (70% non-White). The average Mini-Mental State Exam score (ranging from 0 to 30) at enrollment was 17.1 (*SD* = 7.7). A little over one-third (34%) lived at home alone. Over the first 12 months after enrollment, MIND at Home participants received, on average, three contacts per month through in-person, phone, and email contacts. Table 1 highlights that the dominant method of communication was via phone, with approximately 57% of all contact related to having a phone conversation or leaving a voicemail. Most contacts were between the MCC and the informal caregiver. Twelve percent of all contacts were in-person, averaging approximately one in-person visit per participant every 3 months.

**Table 1.** Frequency and Percent of Contacts by MIND at Home Team with 342 Participants in First 12 Months of Intervention

Contact type	Total number of contacts	Percent of total contacts	Annual number of contacts per person
Phone	4,573	37.79	13.37
Phone Left Message	2,393	19.77	7.00
In-Person	1,474	12.18	4.31
Email	1,218	10.06	3.56
Consultation	1,077	8.9	3.15
Mail	819	6.77	2.39
Other^a^	548	4.53	1.60
	11,554	100	

*Note*: SOURCE Authors’ analysis of the MIND at Home Dementia Care Management System 2.0.

^a^Other category includes fax, text, research activities for the PWD, telehealth, and quality improvement reviews.

Cost of care management was modeled using the average time spent across the first 12 months. Table 2 reports the average time per participant per month by each provider and the average travel time per month. MCC contributed the greatest time to the care coordination of PWD and their care partners, providing over an hour and a half of time per dyad per month (support required by both the PWD and their caregiver). Despite not being a RN-led model, RNs are still contributing on average 25 min per dyad per month to the program, mostly through the office-based weekly collaborative case meetings. Contributions of geriatric psychiatrists and MCC supervisors provide approximately 17 and 12 min, respectively, per dyad per month. As providers are often reimbursed in fifteen-minute intervals, this equates to 2.75 hr per dyad per month of reimbursable time. Using local salaries, the cost of providing this care is $110 per dyad per month. There were no differences in time spent with participants who were dually-eligible compared to those who were Medicare only.

**Table 2.** Calculation of Monthly Care Coordination Activities Per Person

	MCC time	MCC supervisor time	RN time	MD time	Travel (miles)	Total
Average First 12 months (minutes)	91.24	11.61	24.10	2.31	16.68	
Average Time (hours rounded up to nearest 0.25)	1.75	0.25	0.5	0.25		
Rate (Salary and Fringe^a^)	$23	$32	$53	$106	$0.55	
Cost per participant per month	$40	$8.00	$26.50	$26.50	$9.17	**$110**

*Note*: Authors’ analysis of the Johns Hopkins Dementia Care Management System 2.0.

MCC = memory care coordinator; MD = medical doctor; RN = registered nurse.

^a^Salary and Fringe information for the MIND at Home project are derived from actual salary information.

Table 3 shows the results of the Medicaid spending difference-in-differences analysis between MIND at Home participants and the propensity score matched comparison group. Medicaid expenditures of participants grew 3.27% per quarter in the five quarters after initiation of MIND at Home services compared to the 2-year preintervention baseline, while expenditures of the matched comparison group rose by 4.39%. That is, there is a 1.12 percentage point faster increase in spending per quarter among the comparison group compared to participants. Nearly all of the savings come from slower growth in Medicaid hospital inpatient spending and long-stay facility spending for participants compared to the comparison group. As given in Table 3, hospital inpatient spending for participants grew 8.8% per quarter in the first five quarters following implementation of MIND at Home compared to 13.9% for the comparison group, a difference of 5 percentage points per quarter. Long-stay facility spending for participants grew at 38% per quarter compared to 46% for the comparison group, a difference of 8 percentage points per quarter. Home and community-based services and physician services grew slightly faster for participants than the comparison group reflecting better access to these important basic services. Analysis of mean total spending may mask substantial variation within a population. A sensitivity analysis of the difference between participant and control median total Medicaid spending showed more significant differences than those reported in the mean total spending analysis. Median total spending growth increasing 11.9% faster per quarter among the comparison group than participants. Stratifying by dual status showed the substantial growth in Medicaid spending among the comparison group occurring among Qualified Medicare Beneficiaries rather than full duals (see Supplementary Table 2).

**Table 3.** Results of Difference-In-Differences Analysis of Total Medicaid Spending Between MIND at Home Participants and Matched Comparison Group and by Service Type

		Preintervention period	Intervention period (Q1-Q5)	Difference pre-post	% Difference pre-post	% Difference per quarter	Difference-in-differences per quarter
Total Spending	Comparison Group	$3,793	$5,790	$1,997	53%	4.39%	
	Participants	$3,427	$4,771	$1,343	39%	3.27%	1.12%
Medicaid Spending by Service Type							
Inpatient	Comparison Group	$131	$348	$217	166%	13.86%	
	Participants	$113	$234	$120	106%	8.83%	5.04%
HCBS	Comparison Group	$2,894	$3,852	$957	33%	2.76%	
	Participants	$2,583	$3,479	$897	35%	2.89%	-0.14%
Outpatient	Comparison Group	$145	$152	$7	5%	0.38%	
	Participants	$256	$228	-$28	-11%	-0.92%	1.30%
Physicians	Comparison Group	$233	$272	$40	17%	1.43%	
	Participants	$166	$232	$66	40%	3.33%	-1.91%
LTC	Comparison Group	$133	$870	$737	552%	46.00%	
	Participants	$59	$330	$270	455%	37.93%	8.06%
Special Programs^a^	Comparison Group	$218	$283	$65	30%	2.51%	
	Participants	$198	$253	$55	28%	2.33%	0.18%

*Note*: Authors’ analysis of the Maryland Medicaid Claims.

HCBS = home- and community-based services; LTC = long-term care.

^a^Special programs refer to laboratory services, radiology, and durable medical services and equipment.

**Figure 1.**
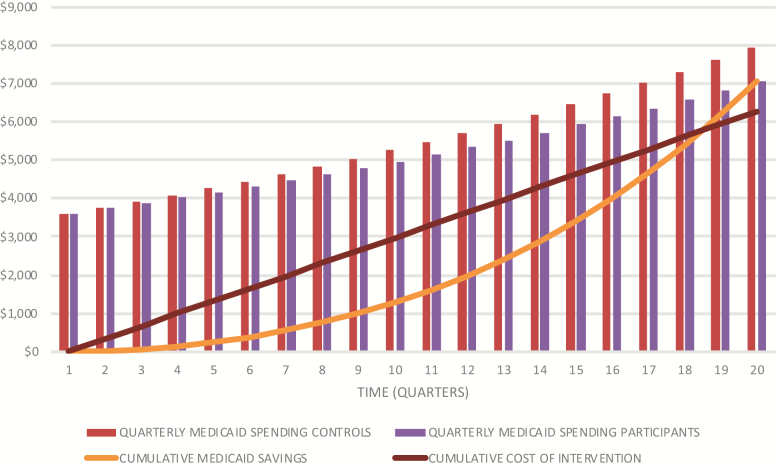
Return on investment over 5-year period comparing cumulative cost savings between participants and the matched comparison group Medicaid spending and the cumulative cost of the intervention.

We use this analysis to model a projection of Medicaid expenditures for 20 quarters, or 5 years. If these trends were to continue, at the end of 5 years, Medicaid expenditures per quarter for participants is projected to be an average of $7,062, compared to $7,937 for the matched comparison group. Cumulative savings over the first 5 years would be $7,052, assuming an average of the preintervention Medicaid spending ($3,610) between the comparison group and participants (Figure 1). Net of the cost of the 5-year MIND at Home intervention, 5-year Medicaid savings are estimated at $782 per beneficiary, a 1.12-fold return on investment. However, it should be recognized that these projections are based on a very limited follow-up period and may change substantially (higher or lower) as participants are tracked over a longer period of time.

## Discussion and Implications

Given the complexity and diversity of needs in dementia, dementia care coordination offers much promise in improving outcomes for both the person with dementia and the care partner (Samus et al., 2014) Based on the analysis of Medicaid spending, the MIND at Home program slowed and redistributed spending away from inpatient and long-term nursing facility spending, and towards physician and home and community based services. Although the Medicaid spending analysis only reflects the proportion of services that Medicaid covers, these findings are similar to those reported in an earlier RCT of this intervention that those who participated in the MIND at Home program experienced statistically significant delay to all-cause transition to home (including transition into long-term nursing facilities) or death (Samus et al., 2014) and increased use of home and community based services, and outpatient dementia/mental health visits (Amjad et al., 2018). Unlike this analysis of Medicaid spending, the earlier study found no statistically significant differences in inpatient care or total outpatient care. This may be a reflection of the study population differences across the two studies with this analysis reporting outcomes exclusively for low-income older adults enrolled in the Medicaid.

The MIND at Home intervention achieves these outcomes through five core principles of comprehensive dementia care management. First, its focuses on home-based dementia care which allows for the identification and assessment of a much broader range of care needs that take into account the living environment, safety, and social determinants of health compared to office-based assessments with more limited scope. Second, it is team-based, recognizing the diverse challenges and needs of PWD, and the different and valuable contributions of a diverse team of care providers to addressing these needs. In particular, it draws on the growing evidence that community health workers represent a unique and valuable public health intervention approach that bridges medical organizations, community resources, patients, and families. Third, it is a high-touch intervention, supporting and empowering PWD and their informal caregivers to manage and coordinate their care. MCCs, on average, contacted the MIND at Home participant and care partner three times a month and visited them in person at least once every 3 months. Fourth, it offers unique opportunities for increasing and expanding workforce capacity potential. Finally, it offers a low-cost solution to dementia care coordination, an issue that has plagued many care coordination programs that have aimed to provide high-touch support in the past (McWilliams, 2016). With approximately 3.6 million PWD living at home (two-thirds of 5.4 million), the potential cost savings of providing a high-touch, low-cost care coordination model is substantial.

This estimation of the cost of the MIND at Home intervention has some important limitations. First, generalizability is limited because of the study sample accrual methods, which was not a probability sample and represented an urban and suburban catchment area of predominantly dually eligible (full, partial), as well as some Medicare-only beneficiaries with dementia in the Baltimore and surrounding suburbs in Maryland and the District of Columbia. We therefore cannot assume that the time spent coordinating the care of these individuals is representative of all PWD. The Medicaid spending data is limited to the population not enrolled in managed care (Medicare Advantage) therefore findings may not be generalizable to the Medicaid managed care population. The representativeness of the sample may also be affected by selection bias as PWD and their care partners had to be willing to participate in a study. Further, in developing the propensity-matched comparison group, we were limited to matching variables available in the Medicaid claims data which may increase that selection bias. Third, we are dependent on the accuracy of reporting of the MCCs and clinical team for their time over the project. To be as precise as possible we conducted an internal survey of the MCCs and results suggested an underestimation of formal time recorded in the DCMS 2.0 of an average of 30 min per dyad per month. To provide a conservative estimate of the cost of the intervention, we accounted for this additional time in our estimates. Additionally, our ability to measure spending differences and cost savings were limited by small sample size, and to only Medicaid spending that does not reflect the total costs of care experienced by dual-eligibles inclusive Medicare, out-of-pocket spending, and indirect costs related to caregiver work productivity. However, Medicaid spending for Medicare covered services (e.g., inpatient, physician, outpatient, etc.) reflect cost-sharing for service utilization and therefore signal likely greater savings to Medicare in these areas. Finally, in this analysis, we were limited in our ability to assess and account for loss-to-follow-up and to distinguish whether the cause of loss-to-follow-up was due to death, disenrollment from Medicaid, change in status to premium-only Medicaid eligibility, or relocation, etc.

## Policy Implications

A major challenge facing this model is the focus on home-based assessments and use of nonclinical workers, given lack of existing reimbursement in Medicare fee-for-service environments to support this nonlicensed but growing workforce and variations in the formalization of the community health worker role and certification requirements (London, Carey, & Russel, 2016). The Centers for Medicare and Medicaid Services (CMS) will reimburse for Chronic Care Management (CCM) services for Medicare beneficiaries with multiple chronic conditions or cognitive assessment and care plan services for beneficiaries with cognitive impairment billed by physicians and nonphysicians (Centers for Medicare and Medicaid Services, 2016). Nonphysician providers for the purposes of providing CCM or cognitive care plan services include physician assistants, nurse practitioners, and clinical nurse specialists. While these services must be billed by the aforementioned providers, they can be furnished by clinical staff. The definition of clinical staff is somewhat vague, which has limited the uptake of billing for these CCM services. CMS has provided some guidance on clinical staff saying that “clinical staff may only be counted if Medicare’s “incident to” rules are met such as supervision, applicable State law, licensure and scope of practice” (Centers for Medicare and Medicaid Services, 2017). Certification and credentialing of community health workers varies greatly by state (London et al., 2016). These requirements for licensure and state law place barriers to growing the dementia care workforce and suggest that to provide this support effectively, under the supervision of a physician, requires significant training. And yet, in the absence of an affordable, high-touch, care management program, informal caregivers with little to no training are expected to meet the various needs of the person with dementia and navigate the fragmented systems.

Outside of fee-for-service Medicare, in a capitated environment, there is greater flexibility to engage with community workers to provide care management services and the incentives for reducing inpatient and institutional care are better aligned. Whether it be within Accountable Care Organizations (Davis, Willink, & Schoen, 2016), Medicaid Managed Care Organizations, or in Medicare Advantage plans (Willink and DuGoff 2018), providing access to dementia care management services at a low cost has the potential to create significant downstream savings of reduced hospitalizations or emergency department visits. Medicaid Managed Long-Term Services and Supports (MLTSS) plans and Dual Eligible Special Needs Plans that are accountable for institutional care (Lewis, Eiken, Amos, & Saucier, 2018) would be well positioned to implement the low-cost, MIND at Home program that has been shown to delay nursing home placement by approximately 9 months and would likely have the staffing and member assessment infrastructure (e.g., engagement of Community Health Workers and Nurse managers) to implement the model with only mild disruption to workflow processes (Samus et al., 2014).

The MIND at Home program offers a pragmatic and low-cost approach to fill the gap between the existing, predominantly medical, approach to dementia care. CMS should consider expanding their definition of clinical staff able to be reimbursed for CCM and cognitive care plan services under the supervision of physicians and nonphysicians to address the cost challenges of care coordination as well as workforce challenges to address the high needs of many older adults, particularly those with dementia.

## Supplementary Material

igz051_suppl_Supplementary-Material
