# Supplementary material for: Cost-Effective Care Coordination for People With Dementia at Home
Source: Innov Aging. 2020 Jan 1;4(2):igz051. doi: 10.1093/geroni/igz051 (PMC6938464; doi:10.1093/geroni/igz051)
Supplement: igz051_suppl_Supplementary-Material [file igz051_suppl_supplementary-material.docx]

SUPPLEMENTAL MATERIAL

Supplemental Table 1: Results of Propensity Score Matching

|  | **Full Medicaid Eligibility** | | | **Qualified Medicare Beneficiary (QMB)** | | |
| --- | --- | --- | --- | --- | --- | --- |
|  | **Treatment** | **Comparison Pool** | **Match (3:1)** | **Treatment** | **Comparison Pool** | **Match (3:1)** |
|  | **n=70** | **n=3722** | **n=210** | **n=50** | **n=1106** | **n=150** |
| **Demographics** | | | | | | |
| Average Age | 80.9 | 73.7 | 81.2 | 79.6 | 73.6 | 79.8 |
| Percent White | 19.5% | 34.3% | 18.2% | 22.2% | 36.9% | 24.1% |
| Percent Male | 20.8% | 33.9% | 16.9% | 24.1% | 28.7% | 22.2% |
| **Service Flags and Conditions** | | | | | | |
| Percent Medicaid HCBS | 49.4% | 47.1% | 56.3% | 0.0% | 1.1% | 0.0% |
| Percent Medicaid Nursing Facility | 7.8% | 13.5% | 9.5% | 14.8% | 13.8% | 14.8% |
| Percent Medicare Emergency Room | 48.1% | 55.1% | 51.1% | 74.1% | 63.0% | 71.0% |
| Percent Medicare Inpatient | 27.3% | 34.9% | 28.6% | 46.3% | 39.5% | 40.1% |
| Number of Chronic Conditions | 3.7 | 4.6 | 4.0 | 4.7 | 4.5 | 4.8 |
| **Combined Costs** | | | | | | |
| Average Combined PMPM | $3,042 | $3,853 | $3,313 | $1,635 | $2,176 | $1,746 |

Notes: Authors’ analysis of Medicare Claims Data and Medicare Master Beneficiary Summary File; HCBS: Home and Community Based Services, PMPM: Per Member Per Month

Supplemental Table 2: Stratified analysis of the difference-in-difference analysis by Medicaid status

|  |  | Pre-intervention Period | Intervention Period (Q1-Q5) | Difference Pre-Post | % Difference Pre-Post | % Difference Per Quarter | Difference-in-Differences Per Quarter |
| --- | --- | --- | --- | --- | --- | --- | --- |
| Total Population | | | | | | | |
| Total Spending | Comparison Group | $3,793 | $5,790 | $1,997 | 53% | 4.39% |  |
|  | Participants | $3,427 | $4,771 | $1,343 | 39% | 3.27% | 1.12% |
| Full Duals | | | | | | | |
| Total Spending | Comparison Group | $6,370 | $7,947 | $1,577 | 25% | 1.90% |  |
|  | Participants | $5,425 | $6,832 | $1,407 | 26% | 2.00% | -0.09% |
| Qualified Medicare Beneficiaries | | | | | | | |
| Total Spending | Comparison Group | $634 | $1,575 | $941 | 148% | 11.42% |  |
|  | Participants | $874 | $925 | $51 | 6% | 0.45% | 10.97% |
